# Electronegativity-Inspired Multidimensional and Complex Representations for Modeling Chemical Environments in Materials

**DOI:** 10.1021/acs.jpclett.6c01562

**Published:** 2026-07-28

**Authors:** Youngjin An, Soyeon Jeon, Da Bean Han, Hyun Woo Kim

**Affiliations:** † Department of Chemistry, 65419 Gwangju Institute of Science and Technology, Gwangju, 61005, Republic of Korea; ‡ Center for Quantum Technology, Korea Institute of Science and Technology, Seoul 02792, Republic of Korea

## Abstract

Materials discovery is increasingly facilitated by data-driven approaches, which necessitate accurate descriptors of chemical bonding. Conventional scalar electronegativity, one of the essential descriptors, cannot capture the complex electronic environments in materials. Here, we introduce multidimensional electronegativity-inspired representations for most elements and extend them to complex-valued forms to more effectively describe the local chemical environments. Starting from Pauling’s definition based on bond dissociation energy, we optimize these vectors using the energy stabilization associated with material formation. Inspired by Mulliken’s definition, we further incorporate ionization energy and electron affinity into the real and imaginary components of the vectors. Using crystal graph convolutional neural networks (CGCNNs) and a complex-valued variant, we demonstrate that these descriptors more accurately capture atomic interactions in local environments, leading to improved convergence behavior. Our results highlight that multidimensional and complex-valued representations are effective descriptors for modeling chemical environments in materials and provide a promising approach to materials discovery.

## Graphical abstract

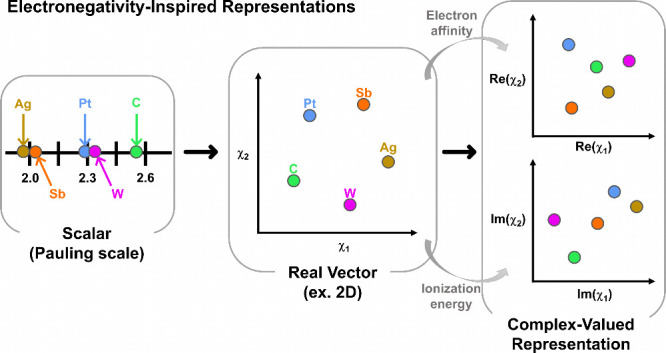

In materials chemistry, the development of descriptors that capture chemical bonding and electronic structure has become increasingly important for understanding and predicting the material properties. To this end, Pauling introduced the concept of electronegativity as a quantitative measure of how strongly an atom attracts electrons in a chemical bond, establishing a thermochemical scale based on bond dissociation energies.^1^ This scale has been widely adopted in recent studies to rationalize bonding and property trends in diverse material systems.^2,3^ Subsequent formulations refined and reinterpreted electronegativity from complementary perspectives: Mulliken defined it in terms of ionization energy and electron affinity;^4^ Sanderson emphasized electronegativity equalization as a framework for charge redistribution;^5^ Allred and Rochow related it to effective nuclear charge and atomic size;^6^ and Allen proposed a valence-based definition derived from the average energy of valence electrons.^7^ Collectively, these approaches established electronegativity as a central descriptor of bond polarity, charge distribution, and broader chemical trends.^8^ Despite its broad utility, electronegativity remains a single scalar quantity assigned to each atom and, therefore, cannot explicitly capture environment-dependent charge redistribution arising from variations in coordination or local chemical surroundings. Consequently, scalar descriptors provide only limited insight into the complex electronic interactions that govern the chemical bonding in materials.

To address these limitations, electronegativity can be extended beyond that of a static atomic quantity. Bultinck et al. showed that within the electronegativity equalization framework, electronegativity behaves as a system-dependent property that is redistributed and equalized across atoms in response to their chemical environment.^9^ Racioppi et al. further developed an *in situ* electronegativity concept that accounts for charge redistribution in extended systems, enabling electronegativity to dynamically adapt to the local bonding and electronic environment.^10^ More recently, representing electronegativity as a multidimensional vector in molecular systems has enabled a more refined classification of the bonding environments.^11^ These developments highlight the potential of environmentally aware descriptors to capture the electronic complexity of inorganic materials.

Building on this idea, various approaches to material representation have been developed to improve property prediction. These approaches can be broadly classified into descriptor-based and structure-based methods. Descriptor-based methods include data-driven workflows for catalyst discovery^2,12–14^ as well as structure-free encodings that follow machine-readable representation principles.^15,16^ In contrast, structure-based methods learn structure–property relationships directly from atomic structures, often using deep learning models.^17,18^ Within structure-based methods, graph-based architectures are particularly prevalent for predicting properties from crystal structures.^19^ Crystal graph convolutional neural networks (CGCNN) represent a crystal as a graph, with atoms as nodes and interatomic distances as edges, enabling the model to learn local atomic environments directly from structure.^20^ Subsequent developments incorporated higher-order geometric interactions, including bond-angle information through line graph neural networks,^21,22^ as well as more general graph-network formulations such as MEGNet, which incorporate global state variables.^23^ More recent efforts have focused on improving representation learning and training scalability for better generalization across chemically diverse inorganic materials.^24–26^ Additionally, graph-based machine-learned interatomic potentials (MLIPs) have been developed to predict both material energies and atomic forces, enabling atomistic simulations across diverse materials.^22,27,28^

Typically, the node features in CGCNN are based on intrinsic atomic properties such as electronegativity, atomic radius, and atomic weight, which capture general chemical trends across the periodic table. Although these features are informative, they are inherently static and do not explicitly account for electronic redistribution resulting from chemical bonding. Recent studies have employed language model-based descriptors^29–31^ and embeddings extracted via machine learning^32–34^ to improve structural representations. However, these methods are not designed to explicitly capture the electronic aspects of chemical structures. Consequently, although current descriptors effectively represent local atomic environments, they provide limited insight into critical features of chemical bonding.

In addition, most descriptors have been used on an equal footing, restricting the integration of information from multiple chemical spaces. To overcome this limitation, complex-valued spaces have been employed in complex-valued neural networks (CVNNs).^35–37^ CVNNs are widely used in signal processing^38–40^ and quantum physics,^41,42^ where they demonstrated greater expressive power than real-valued counterparts. As complex numbers consist of real and imaginary parts, linear transformations in complex space correspond to two-by-two rotations and scaling operations,^43^ enabling more diverse feature representations. Despite these advantages, the application of CVNNs in chemistry has been overlooked, particularly in predicting material properties. Moreover, their potential for modeling atomic bonding environments and electronic characteristics is not fully understood and remains an area for future exploration.

Here, motivated by previous definitions of electronegativity, we develop material representations using electronegativity-inspired features in real-valued vectors. We further employ complex-valued representations to efficiently describe structure–property relationships in materials. Specifically, we introduce a multidimensional electronegativity descriptor designed to overcome the inherent limitations of conventional scalar electronegativity. Although our approach is not limited by dimensionality and provides electronegativity in Pauling scale as a 1D representation, the 32-dimensional electronegativity-inspired representation (χ_*ML*_^(32)^) demonstrates significant performance improvements through modifications of the atomic descriptor. Furthermore, the tendency of each element to donate or accept electrons can be explained using its electron affinity and ionization energy,^4^ which are incorporated into the real and imaginary components of a complex space within a complex-valued CGCNN (CV-CGCNN). This method improves the accuracy of formation energy predictions by approximately 13% compared to the original CGCNN model. These findings indicate that complex-valued operations extend beyond mere numerical generalization, enabling a more meaningful representation of atomic properties within materials. Overall, this work advances the field of materials informatics by overcoming the limitations of conventional electronegativity and existing atom property-based descriptors with the introduction of χ_*ML*_^(32)^, while highlighting the potential of complex-valued neural networks for improved chemical property prediction.

Pauling electronegativity is defined based on the difference in bond dissociation energies between two atoms, as shown in eq 1:

1 $$f\left(\chi_{i},\chi_{j}\right)={\left(\chi_{i}-\chi_{j}\right)}^{2}=D_{ij}-\frac{D_{ii}+D_{jj}}{2}$$

where *D*_*ij*_ denotes the dissociation energy of the bond between element *i* and element *j*, and *D*_*ii*_ and *D*_*jj*_ are the dissociation energies of the homonuclear bonds of element *i* and element *j*, respectively. This formulation quantifies the relative strength of an *i*–*j* bond compared to the average of the corresponding homonuclear *i*–*i* and *j*–*j* bonds, reflecting how local electronic interactions influence bond stability.

In a crystalline material, however, each atom interacts with multiple neighboring atoms, which makes a two-body description such as eq 1 insufficient. Explaining the many-body and environment-dependent interactions therefore requires a descriptor or modeling framework that extends beyond the pairwise interactions. Graph-based machine-learning models such as CGCNN provide a framework by representing crystal structures as graphs and learning interatomic interactions in a data-driven manner.

Within this framework, the formation energy of a crystal,

2 $$E_{\mathrm{formation}}=E_{\mathrm{total}}-\underset{i}{\sum }n_{i}\mu_{i}$$

can be interpreted as a quantity conceptually analogous to Pauling electronegativity. Here, for each element *i*, *n*_*i*_ denotes its atomic count in the crystal, and μ_*i*_ represents the corresponding reference energy per atom in the standard elemental phase. As eq 1 captures the deviation of a heteronuclear bond energy from the average of the corresponding homonuclear bond energies, the formation energy measures the deviation of the total energy of a crystal from the sum of the energies of its constituent elements in their standard states. In this sense, both quantities reflect how electronic interactions stabilize a system relative to elemental references: Pauling electronegativity at the level of individual bonds, and formation energy at the level of the entire crystal. Thus, the formation energy can serve as a physically meaningful target property for learning multidimensional electronegativity. In this work, we train CGCNN models to predict formation energy, thereby enabling the model to encode local electronic characteristics in crystalline environments that cannot be captured by conventional scalar electronegativity.

Our procedure for optimizing multidimensional electronegativity is illustrated in Figure 1a and consists of the following steps. First, to distinguish atoms within a crystal structure, graphs are constructed using one-hot encoding, without including additional atomic property descriptors. For each atom, the resulting one-hot vector, **x**_*i*_, is passed through a representation learning layer using an MLP with electronegativity-based normalization (Figure S2). The one-hot vector is first transformed by the MLP as **u**_*i*_ = MLP­(**x**_*i*_). The transformed vector is then normalized and rescaled as

3 $$v_{i}=\frac{u_{i}}{\left|u_{i}\right|}\cdot \chi_{\mathrm{Pauling},i}$$

By construction, the norm of our representation reproduces the Pauling electronegativity, χ_*Pauling*,*i*_ of atom *i*, while the individual components of the multidimensional vector are optimized during training. Subsequently, electronegativity-inspired representations are used as inputs to CGCNN for learning the formation energy of crystal structures. During the training process, the model optimizes electronegativity-inspired representations for each atom.

**Figure 1:**
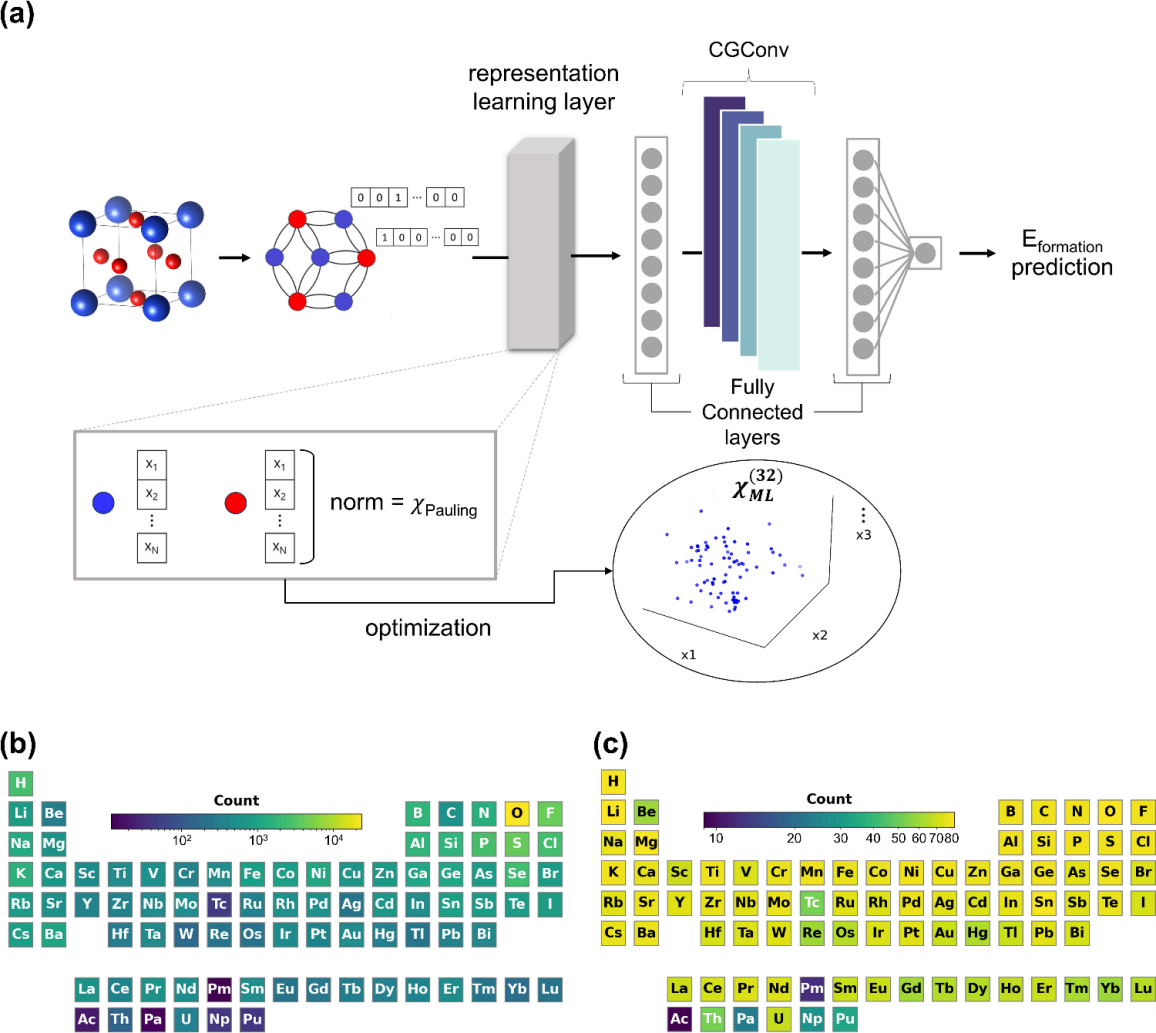
(a) Workflow for optimizing multidimensional electronegativity from a materials structure data set. (b, c) Periodic table showing (b) the number of crystal structures containing each element and (c) the number of distinct interacting elements. The color scheme represents counts in the data set.

In this work, we used a total of 153,087 crystal structures from the Materials Project data set (version 2022.10.28).^44,45^ Of these, 20% were used to optimize the multidimensional electronegativity representations, while the remaining data were randomly split into 60% training, 10% validation, and 10% test sets for model evaluation. This partitioning was repeated 10 times with different random seeds. The model with the lowest validation error was selected, and its final performance was evaluated on the test set to reduce the risk of overfitting. As shown in Figure S3, the validation loss decreased in parallel with the training loss and remained relatively stable throughout training, suggesting limited overfitting under the present training protocol. Figures 1b and c illustrate the elemental distributions in the data set used in this work. Specifically, Figure 1b shows the number of crystal structures containing each element, indicating that most elements appear in approximately 5,000–7,000 structures. Figure 1c presents the number of distinct elements that each element interacts with in the crystal structures, showing that most elements, except for a few rare ones, interact with many other elements. Overall, these distributions indicate that the data set spans a wide range of chemical environments and atomic combinations. All results were obtained from ten independent optimizations using different random initializations.

The dimensionality of electronegativity-inspired representations is essential for balancing effectiveness and physical relevance. In general, increasing the dimensionality improves the representation of atomic electronic properties and can enhance model performance. However, as the proposed representation is defined with a fixed vector norm, excessively high dimensionality may introduce redundant or weakly informative components, leading to a more diffuse representation. Consequently, selecting an appropriate dimensionality is essential. As illustrated in Figure 2a, the prediction accuracy for formation energy varies with the dimensionality of electronegativity, with an optimum at 32 dimensions and reduced sensitivity at higher dimensionalities. As shown in Figures S4–S6, the 32-dimensional descriptor exhibits a more balanced correlation pattern with conventional atomic features than the 16- and 64-dimensional descriptors. These results indicate that a 32-dimensional representation can effectively capture local coordination environments and interatomic interactions within crystal structures while retaining physically relevant relationships with elemental properties.

**Figure 2:**
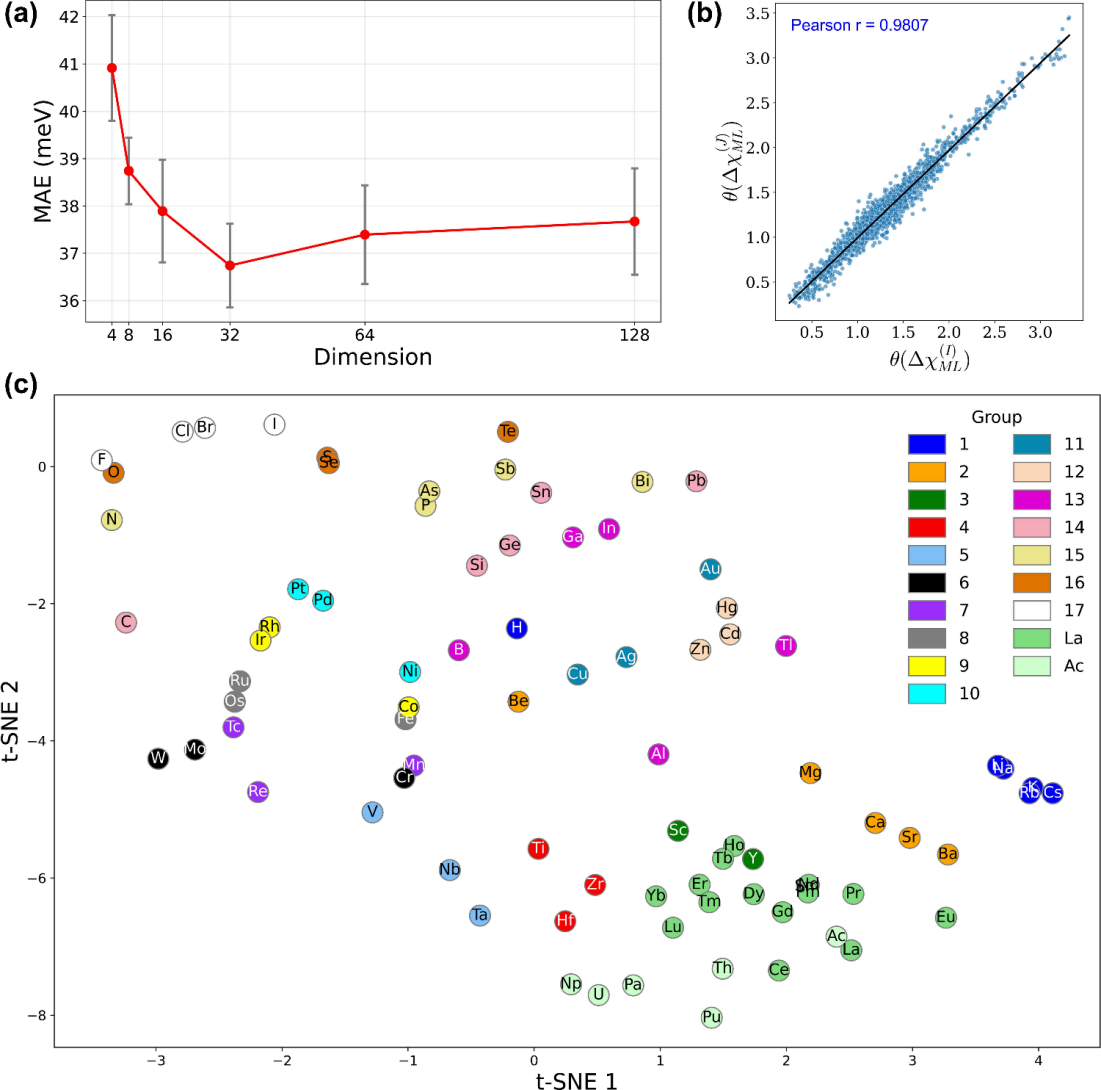
Analysis of electronegativity-inspired representations. (a) Effect of descriptor dimensionality on model performance. (b) Relative angles between difference vectors of representations across different initializations, where *I* and *J* denote initialization indices. (c) Two-dimensional t-SNE projection of χ_*ML*_^(32)^.

Machine learning models may exhibit stochastic variations in embeddings across different iterations due to random initializations during the training process. The consistency and robustness of χ_*ML*_^(32)^ were assessed by examining the correlations across multiple random initializations. As shown in Figure 2b, the differences in χ_*ML*_ from models with different initializations exhibit a high Pearson correlation coefficient, suggesting that χ_*ML*_ shows convergent behavior. In addition, Figure S7 shows that relative angles between χ_*ML*_ also converge consistently, regardless of the initial conditions. The calculation details between learned elemental descriptors are provided in Note S1 in the Supporting Information. In addition, Figure S5 shows that several components of χ_*ML*_^(32)^ exhibit correlations with multiple atomic features, indicating that these components are associated with the physical and chemical properties of elements. Overall, these results suggest that χ_*ML*_^(32)^ serves as a physically relevant, data-driven elemental descriptor rather than a random numerical vector.

To analyze χ_*ML*_^(32)^, t-distributed stochastic neighbor embedding (t-SNE) was employed to project the high-dimensional embeddings into a two-dimensional representation (Figure 2c).^46^ Elements initially represented by one-hot encoding are transformed through the representation learning layer and the CGCNN, resulting in embeddings that capture the chemical environments surrounding each element. As a consequence, elements with similar local chemical environments are located closer to one another in the two-dimensional space generated by t-SNE. By construction, the norm of each χ_*ML*_^(32)^ vector is equal to the Pauling electronegativity of the corresponding element. Thus, the χ_*ML*_^(32)^ vectors follow the general trend of electronegativity values, facilitating the interpretation of chemical bonding patterns. Notably, although transition metal elements have similar electronegativity values, χ_*ML*_^(32)^ shows clear separations that are consistent with differences in their electronic structures, following trends related to the number of *d*-orbital electrons. In addition, χ_*ML*_^(32)^ is reasonably obtained for hydrogen, reflecting its general tendency in crystal structures to exhibit higher electronegativity than metals and to mostly exist in a negatively charged state. In contrast, the original CGCNN descriptor (Figure S8) places elements from Groups 1 to 17 relatively close to each other and groups hydrogen with other Group 1 elements, thereby reflecting only general periodic or block-level trends of the periodic table. These findings indicate that χ_*ML*_^(32)^ captures chemically meaningful elemental properties and overcomes the limitations of conventional atom property-based categorical descriptors by enabling the representation of subtle chemical distinctions.

The performance of χ_*ML*_^(32)^ as a descriptor was evaluated using CGCNN models to predict formation energy, band gap, and Fermi energy. Formation energy is primarily determined by structural characteristics, such as elemental composition, bond strength, and coordination number, whereas band gap and Fermi energy are closely related to electronic features, including the distribution of electronic states and orbital interactions. As illustrated in Figures 3a–c, χ_*ML*_^(32)^ improves the prediction of formation energy, band gap, and Fermi energy by approximately 6%, 12%, and 5%, respectively, compared with the original descriptor. These results indicate that χ_*ML*_^(32)^ effectively captures both atomic arrangements and electronic states within crystal structures. Among the target properties considered in this work, the band gap could be particularly sensitive to the local electronic environment. Furthermore, as shown in Figures 3d–f, χ_*ML*_^(32)^ maintains strong predictive performance relative to other descriptors,^29–33^ demonstrating that sufficient representational capability can be achieved with relatively low dimensionality. Overall, these findings suggest that χ_*ML*_^(32)^ serves as a multidimensional descriptor capable of representing both structural and electronic characteristics.

**Figure 3:**
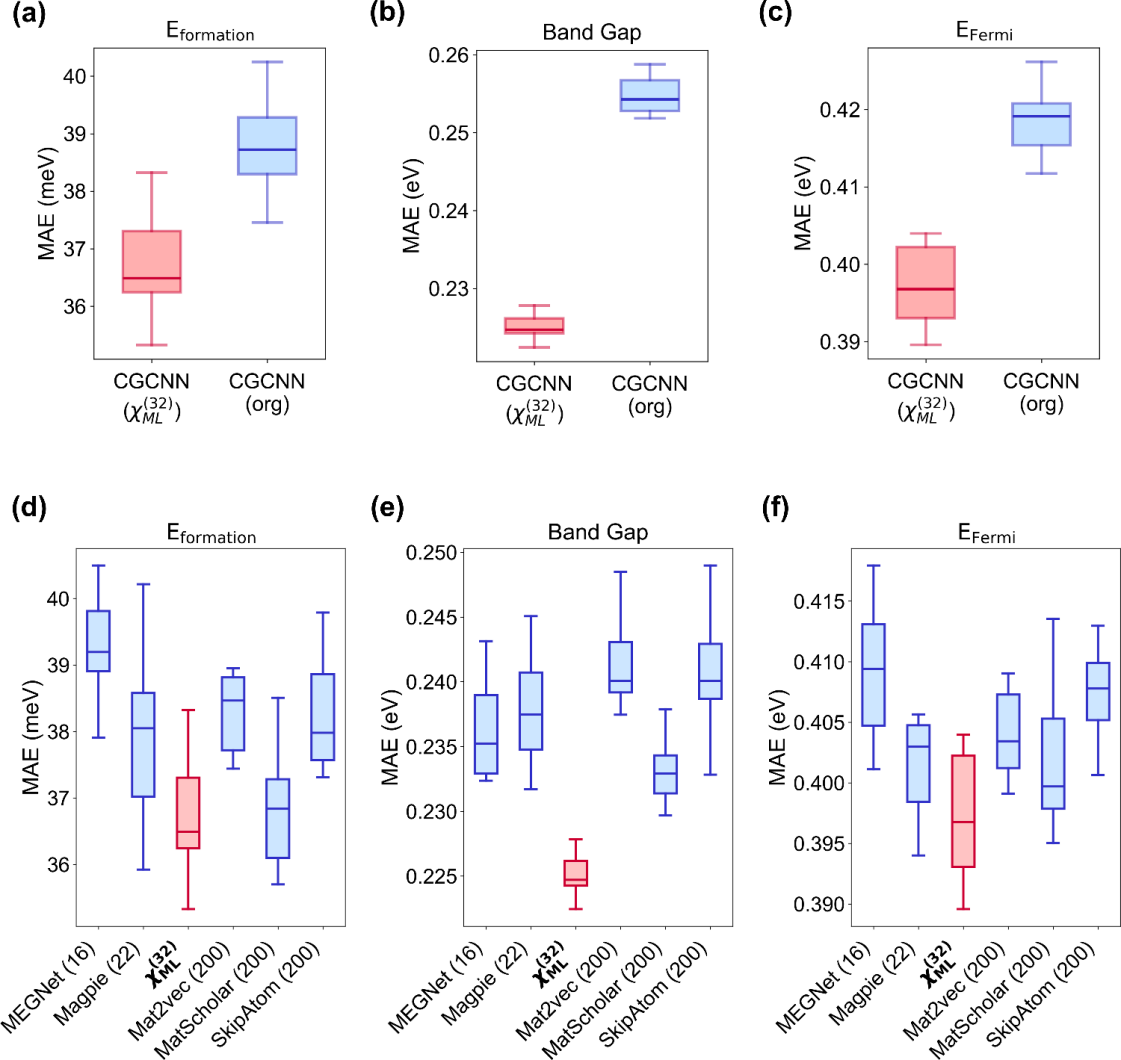
Prediction performance of the CGCNN model using χ_*ML*_^(32)^ compared with the original descriptor for (a) formation energy, (b) band gap, and (c) Fermi energy and compared with other descriptors for (d) formation energy, (e) band gap, and (f) Fermi energy.

**4:**
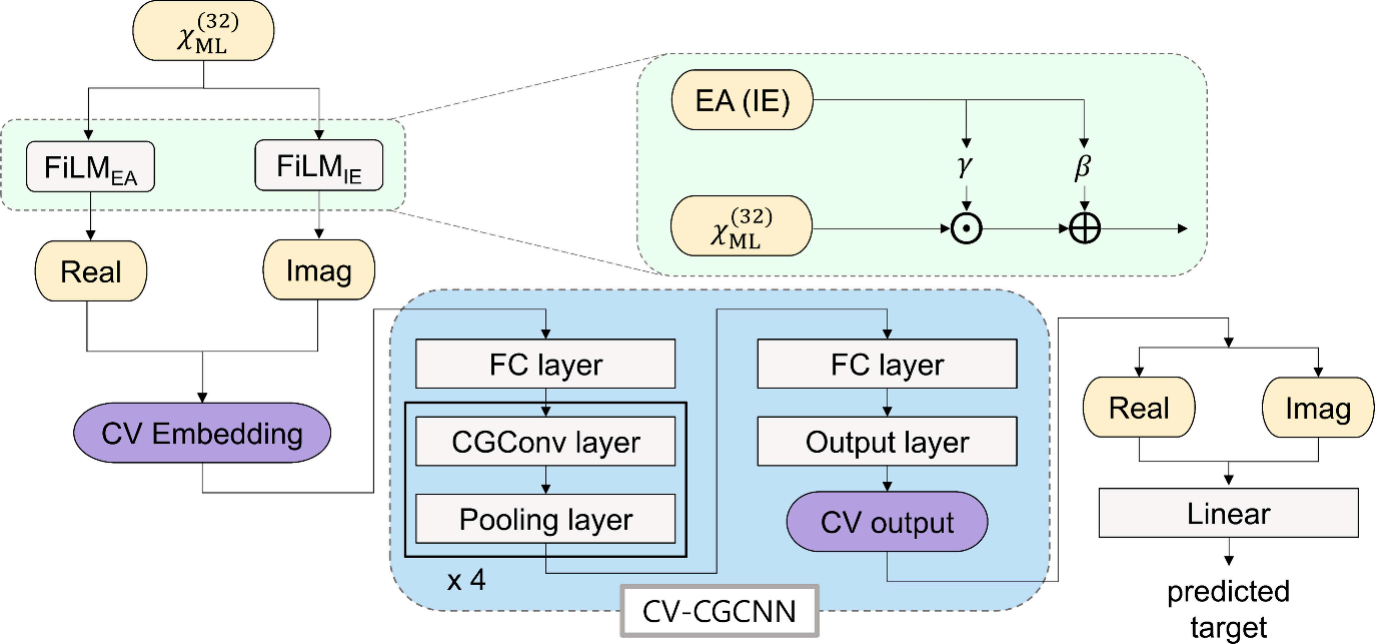
Schematic overview of the complex-valued CGCNN (CV-CGCNN) for property prediction of crystalline materials.

To further improve prediction accuracy using χ_*ML*_^(32)^, we combined the electronegativity definitions of Pauling and Mulliken into a complex-valued representation. In this study, this relationship is considered by incorporating the scalar quantities, electron affinity (EA), and ionization energy (IE) into χ_*ML*_^(32)^ through a feature-wise linear modulation (FiLM) scheme.^47^ The FiLM approach applies learnable scaling (γ) and shifting (β) parameters to each input vector based on scalar inputs, allowing the model to modulate each dimension in a controlled manner. Through this formulation, EA and IE are incorporated into the real and imaginary components, respectively, forming a more effective complex-valued representation of χ_*ML*_^(32)^.

While the original CGCNN model operates with real-valued parameters, its complex-valued extension, named complex-valued CGCNN (CV-CGCNN), can be developed by reformulating its internal operations to handle complex-valued representations. Within the complex-valued fully connected layer of CV-CGCNN, the mapping between an input complex vector **z** and an output complex vector **z**′ is defined as

4 $$z^{\prime }=\mathrm{Wz}+b$$

where **W** and **b** denote the complex-valued weight matrix and the bias vector, respectively. By decomposing the complex vectors into their real and imaginary components, this operation can be represented as the following linear transformation:

5 $$\left[\begin{array}{c} z_{\mathrm{real}}^{\prime }\\ z_{\mathrm{imag}}^{\prime } \end{array}\right]=\left[\begin{array}{cc} W_{\mathrm{real}} & -W_{\mathrm{imag}}\\ W_{\mathrm{imag}} & W_{\mathrm{real}} \end{array}\right]\left[\begin{array}{c} x_{\mathrm{real}}\\ x_{\mathrm{imag}} \end{array}\right]+\left[\begin{array}{c} b_{\mathrm{real}}\\ b_{\mathrm{imag}} \end{array}\right]$$

In this formulation, a complex-valued layer does not merely increase the dimensionality by treating the real and imaginary components independently. Instead, it provides a two-dimensional degree of freedom in which the real and imaginary parts interact. This interaction enhances the representational capacity of the model, making it particularly well-suited for capturing the complex chemical environment in crystal structures with high fidelity.

Complex-valued neural networks employ weights consisting of both real and imaginary components, even when the network depth and architectural structure remain the same. Consequently, for networks of the same depth, CV-CGCNN contains more weight parameters than CGCNN. For a fair comparison, we matched the total number of parameters in both the CGCNN and the CV-CGCNN to approximately 390,000. As illustrated in Figure 5a, the CV-CGCNN (with 394,000 parameters) demonstrates an improvement of approximately 13% in the prediction of formation energy over the original CGCNN (with 398,000 parameters). Moreover, compared with a real-valued CGCNN using χ_*ML*_^(32)^ with a similar number of parameters, CV-CGCNN improves prediction accuracy by approximately 7%.

**Figure 5:**
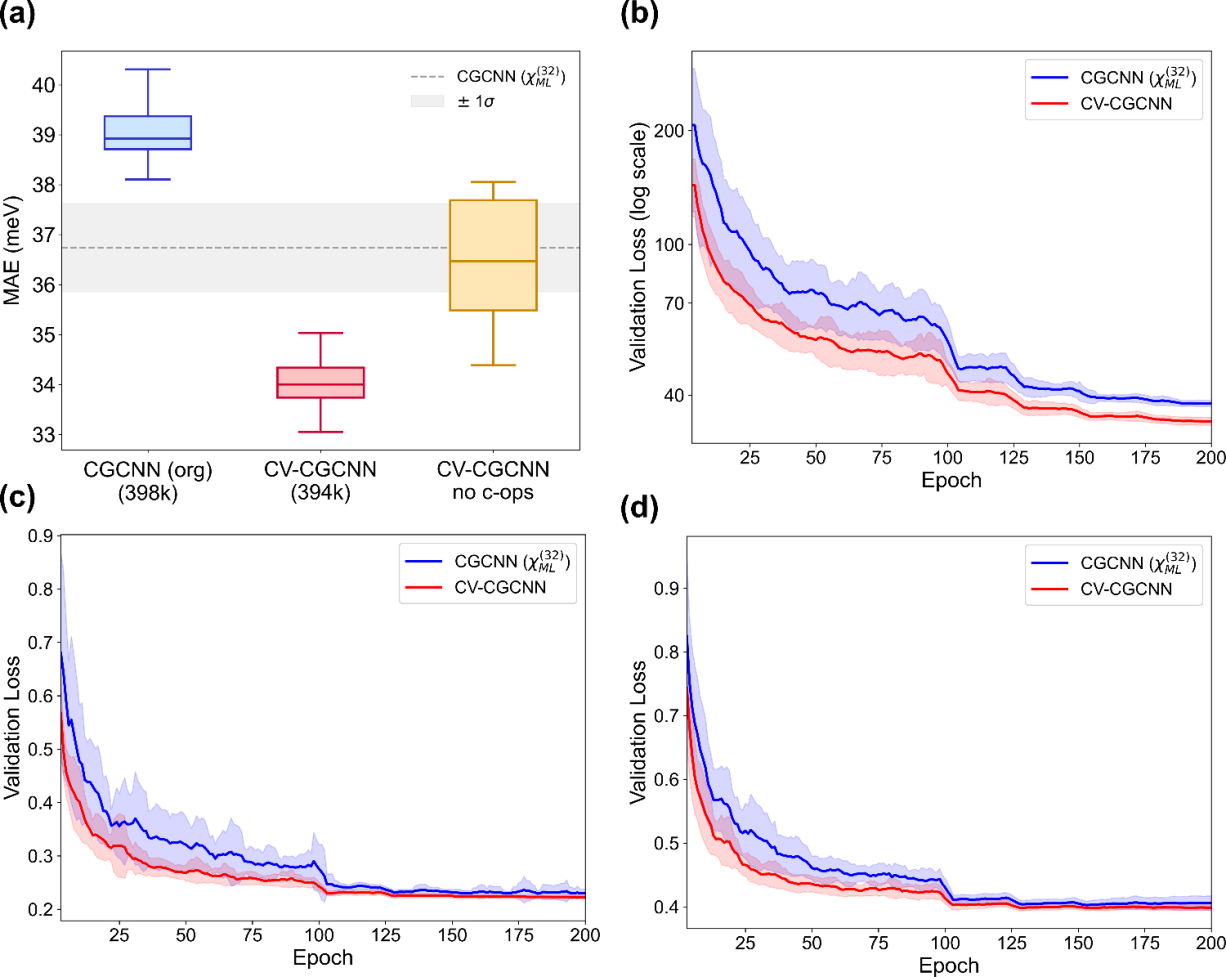
(a) Prediction performance of CV-CGCNN relative to CGCNN with a similar number of model parameters. The yellow bar represents CV-CGCNN without complex operations (c-ops), and the gray baseline corresponds to CGCNN using the χ_*ML*_^(32)^ descriptor. (b–d) Validation loss curves of CGCNN and CV-CGCNN trained using a multistep learning rate scheduler for predicting (b) formation energy, (c) band gap, and (d) Fermi energy.

When complex operations are removed from the complex-valued linear layers, such that the real and imaginary components are processed independently, only limited performance improvements are observed. These results suggest that the observed improvements are not simply due to an increased number of parameters, but rather stem from the complex-valued operations themselves, which enhance the representational capacity. Figure 5b compares the computational cost of the models. Since larger weight matrices must be processed, CV-CGCNN requires approximately 2.2 times more computational time than the original CGCNN. Nevertheless, CV-CGCNN exhibits more rapid convergence during the initial stages of training and consistently achieves lower loss values. Considering both the computational cost and the number of epochs required to reach a comparable validation loss, the total training cost of CV-CGCNN is about 1.4 times that of CGCNN. Although the improvements in band gap and Fermi energy prediction are relatively modest (Figure S9), the training curves in Figure 5c and d show that CV-CGCNN still converges faster than CGCNN with χ_*ML*_^(32)^. These results demonstrate that, despite the increased computational cost, complex-valued representations enable more effective learning of the intricate atomic interactions present in crystal structures. In addition, complex-valued operations can be efficiently implemented on photonic hardware for molecular property prediction, providing faster computation and lower energy consumption.^48^

In summary, we propose an electronegativity-inspired representation, χ_*ML*_^(32)^ within a machine-learning framework, motivated by the insight that the formation energies of crystal structures reflect the complex chemical environments of their constituent elements. By extending electronegativity beyond a scalar quantity, χ_*ML*_^(32)^ yields a more expressive representation that captures electronic differences among elements with higher fidelity. Furthermore, by introducing a complex-valued form for use within the CV-CGCNN framework, we show that the increased representational capacity of complex-valued neural networks enables more effective learning of chemical bonding characteristics. In the future, χ_*ML*_^(32)^ can be used as an alternative or complementary descriptor to conventional electronegativity across various modeling frameworks, enabling improved accuracy in property prediction as well as the investigation of emerging chemical trends. Although this study is primarily focused on property prediction tasks, the development of robust predictive models provides a foundation for generative modeling and emphasizes the potential applicability of χ_*ML*_^(32)^ in crystal structure generation. In addition, the CV-CGCNN approach demonstrates the incorporation of complex-valued operations in chemical modeling. This approach opens up new methodological possibilities, including potential applications in photonic chip-based machine learning.^48^ These advances may enable more accurate and expressive modeling of chemical systems, particularly in fields that have traditionally relied on real-valued representations in machine learning for materials science.

## Supplementary Material



## Data Availability

The source code for multidimensional electronegativity optimization and the implementation of CV-CGCNN is available on GitHub at https://github.com/hwk-grp/en_multi_mater.

## Notes

Detailed information supporting this study (PDF)
