# Supplementary material for: Electronegativity-Inspired Multidimensional and Complex Representations for Modeling Chemical Environments in Materials
Source: J Phys Chem Lett. 2026 Jul 28;17(31):8831–8. doi: 10.1021/acs.jpclett.6c01562 (PMC13455103; doi:10.1021/acs.jpclett.6c01562)
Supplement: Supplementary file 1 [file jz6c01562_si_001.pdf]

Supporting Information

Electronegativity-Inspired Multidimensional and Complex Representations for Modeling Chemical Environments in Materials

Youngjin An[1], Soyeon Jeon[1], Da Bean Han[1], Hyun Woo Kim*[,1, 2]

[1] Department of Chemistry, Gwangju Institute of Science and Technology, Gwangju, 61005, Republic of Korea

[2] Center for Quantum Technology, Korea Institute of Science and Technology, Seoul 02792, Republic of Korea

E-mail: hwk@gist.ac.kr

Parameters	Search Space	Selected Value
batch size	[32, 64, 128, 256]	256
learning rate	[e-4, e-3, e-2]	0.01
weight decay	[0, e-6, e-5]	0
epoch	[100, 150, 200, 250]	200
number of convolution layers	[2, 3, 4, 5]	4
number of hidden layers	[1, 2, 3]	1
convolution dimension	[32, 64, 128, 256]	128

Table S1. Hyperparameter Optimization Details: Search Spaces Based on the Original CGCNN Parameters.

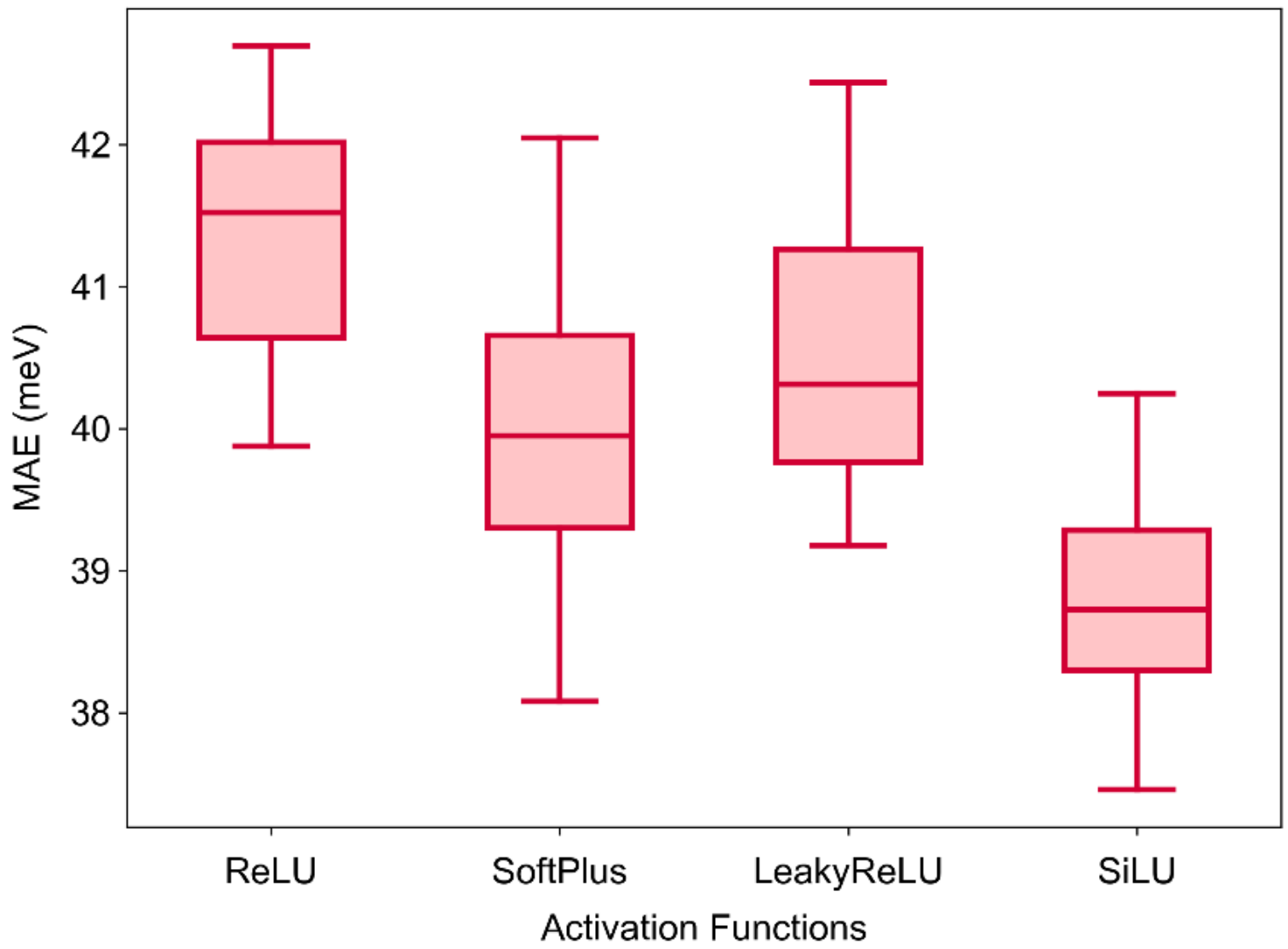

Figure S1. Comparison of formation energy prediction performance across different activation functions.

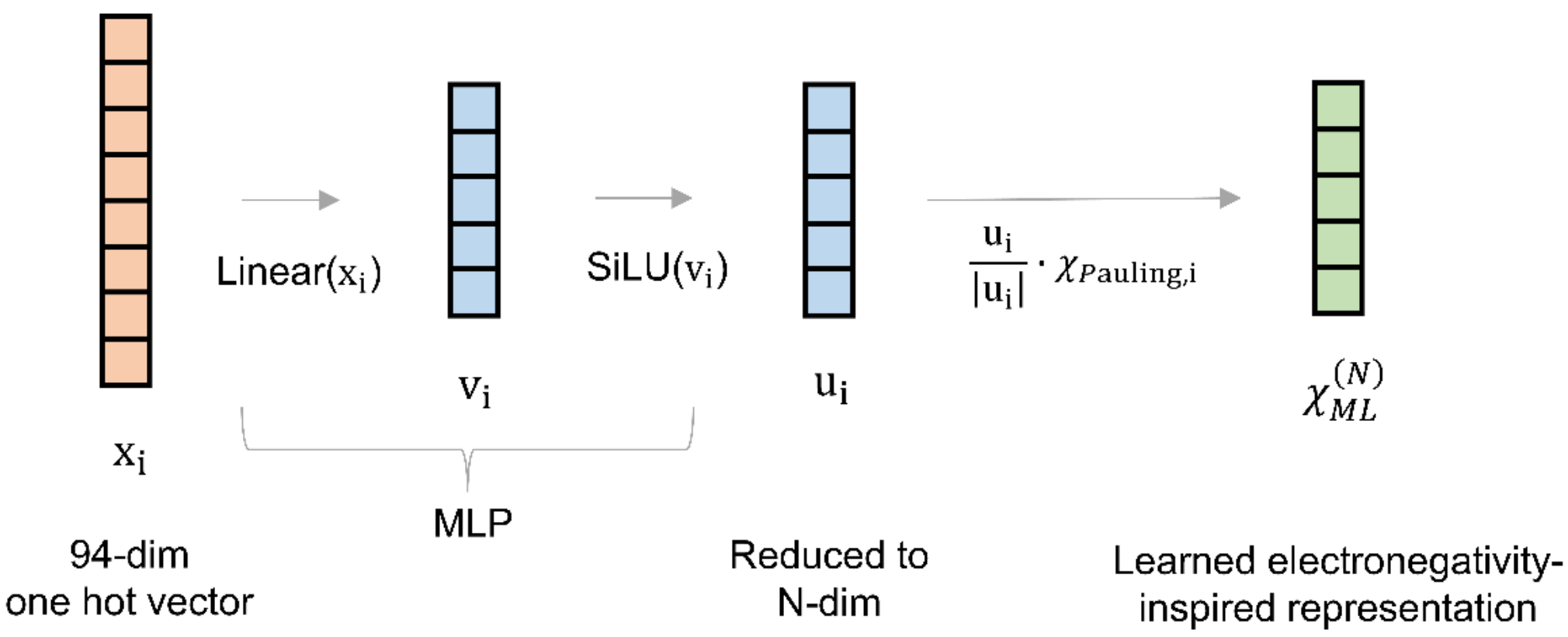

Figure S2. Architecture of the representation learning layer.

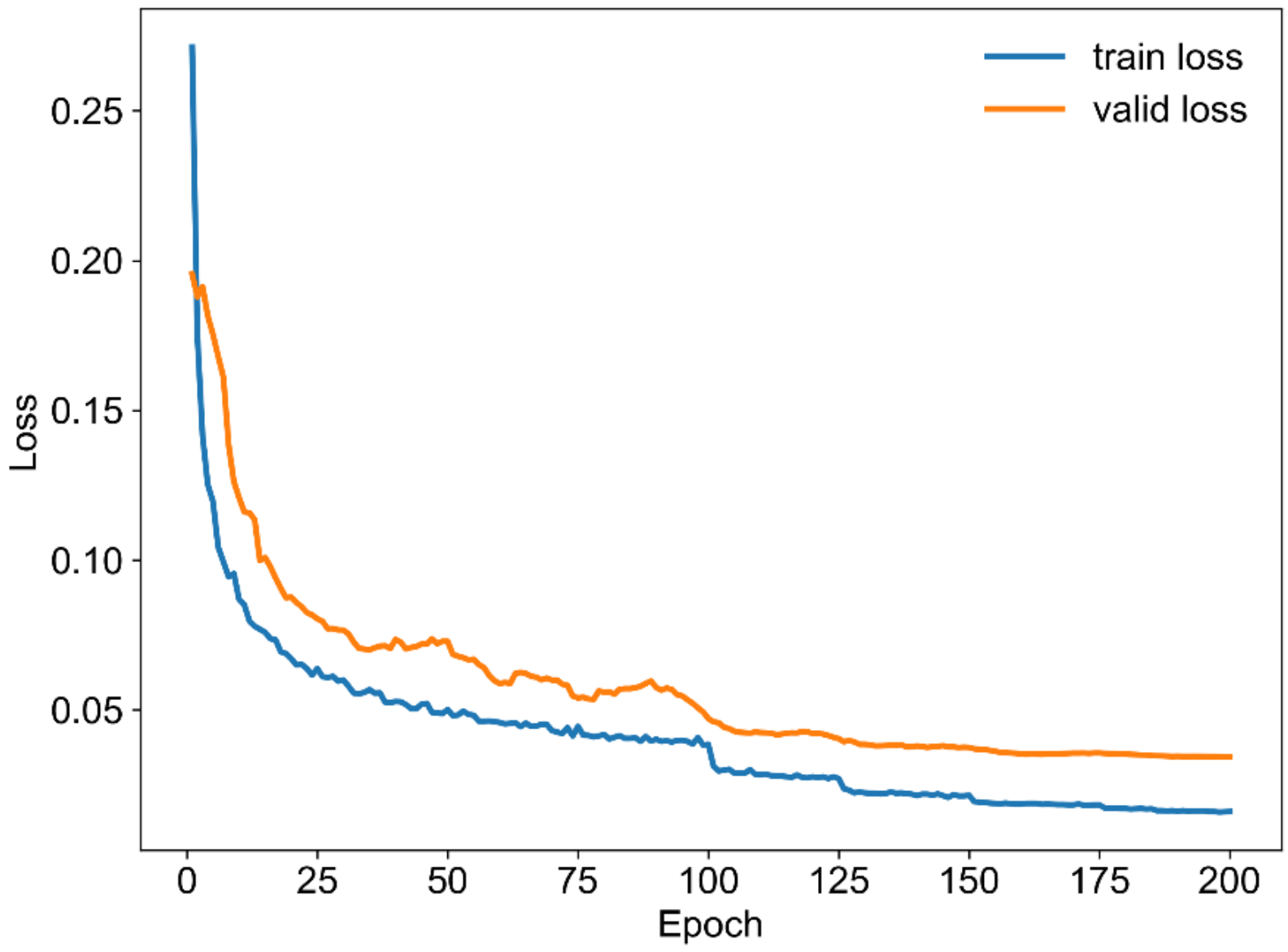

Figure S3. Training and validation loss curves of the CGCNN model for formation energy prediction.

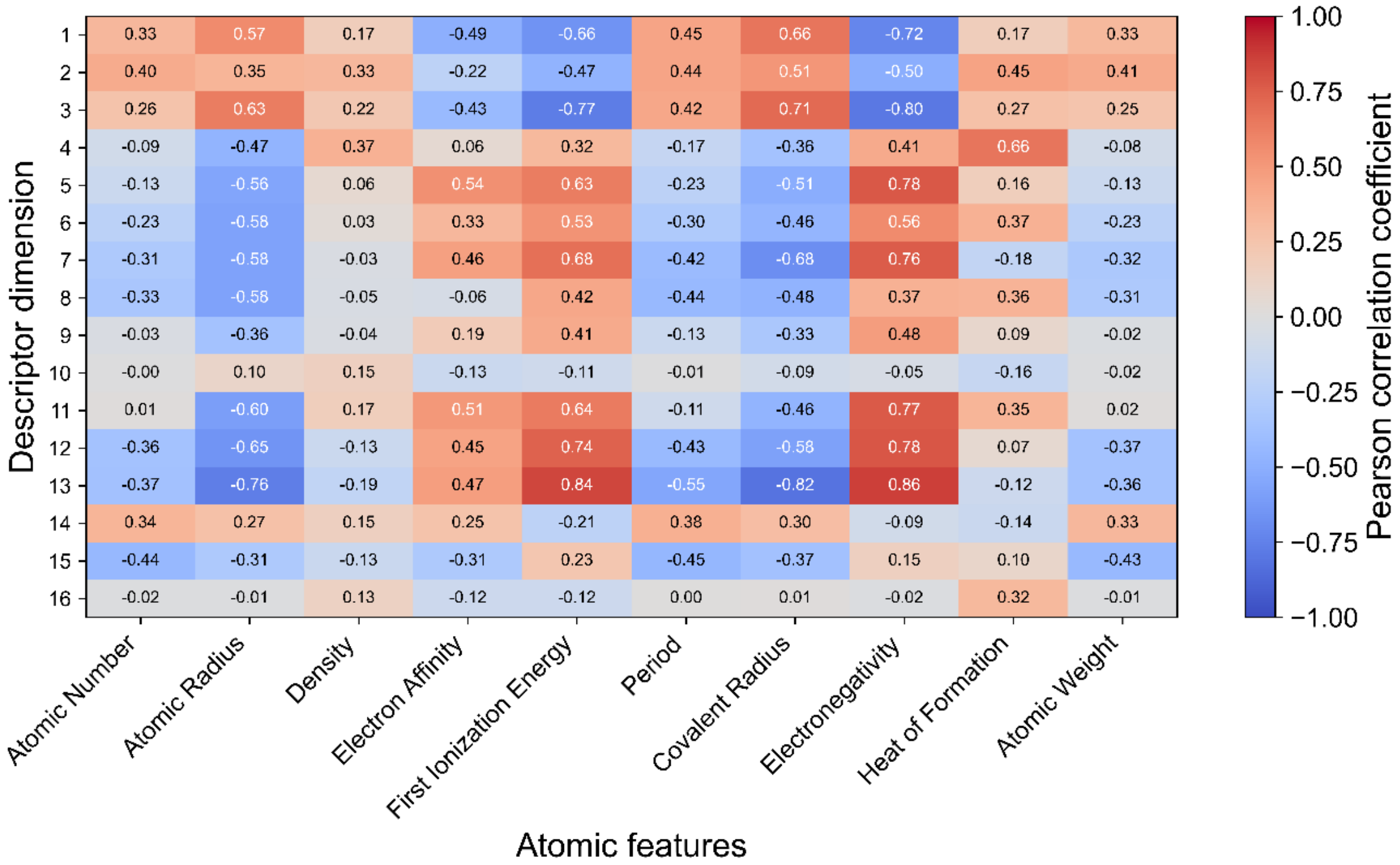

Figure S4. Pearson correlation heatmap of the 16-dimensional descriptor and conventional atomic features.

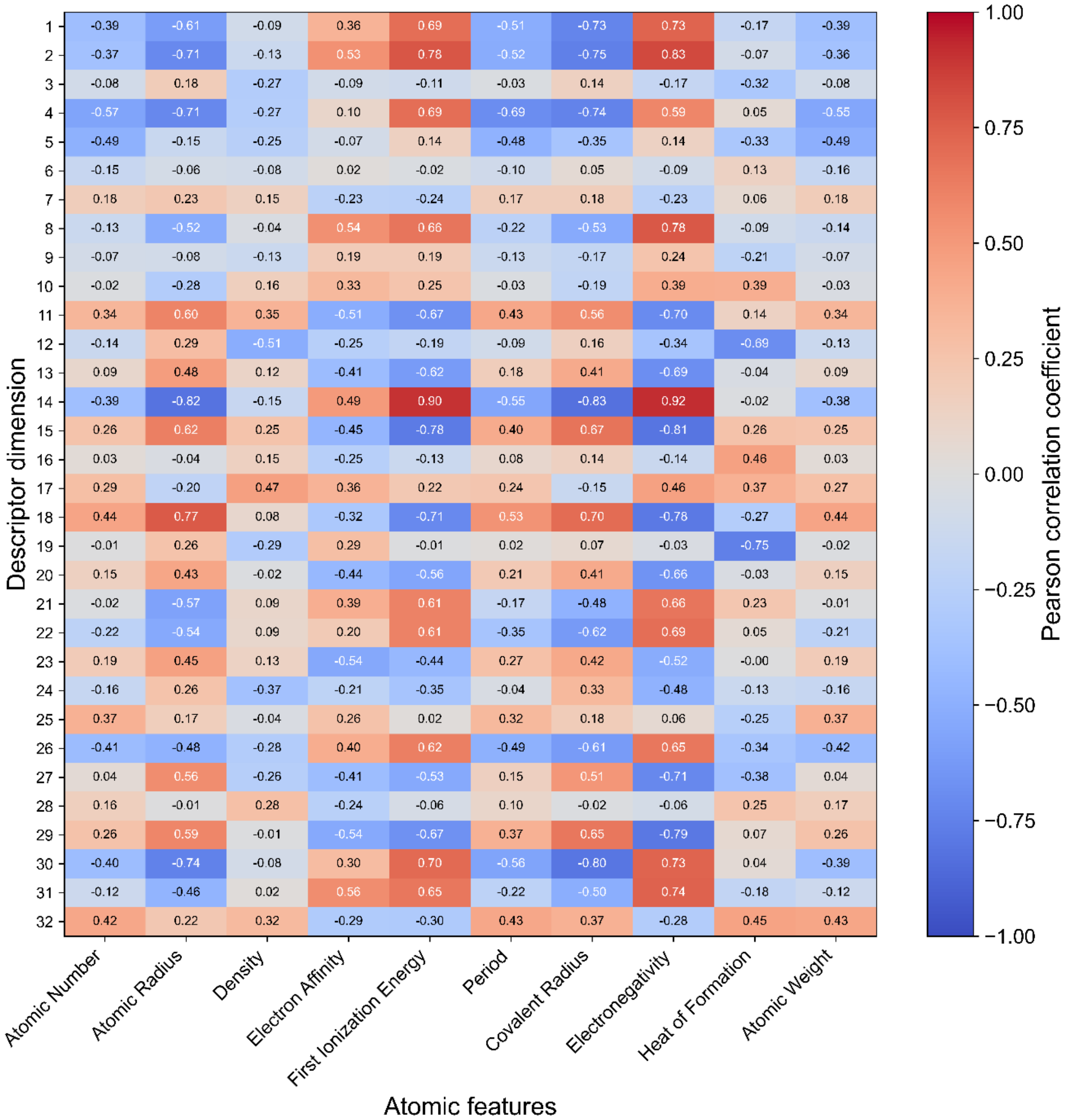

Figure S5. Pearson correlation heatmap of the 32-dimensional descriptor and conventional atomic features.

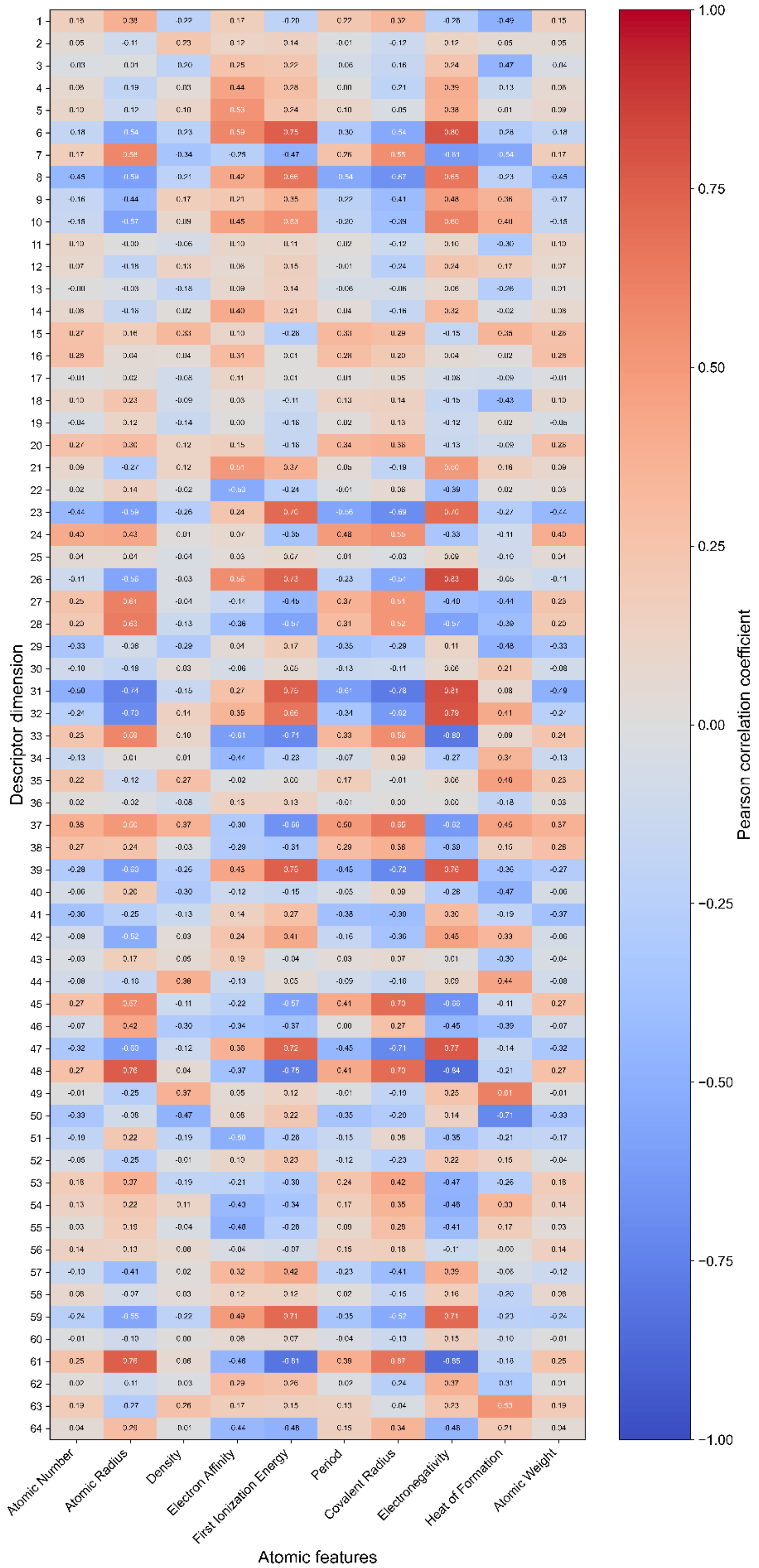

Figure S6. Pearson correlation heatmap of the 64-dimensional descriptor and conventional atomic features.

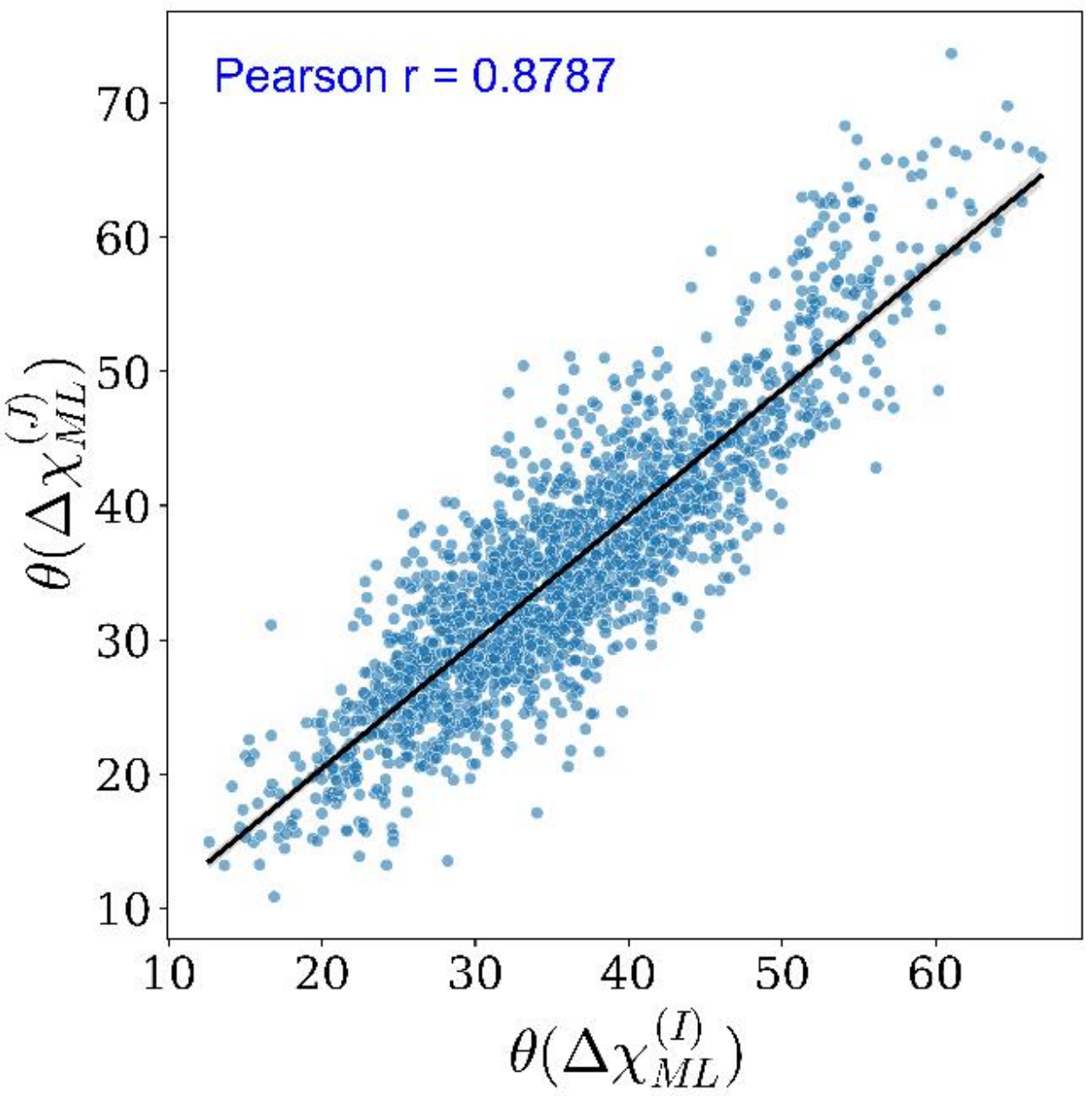

Figure S7. Pearson correlation between relative angles of the learned elemental vectors obtained from models with different initial conditions.

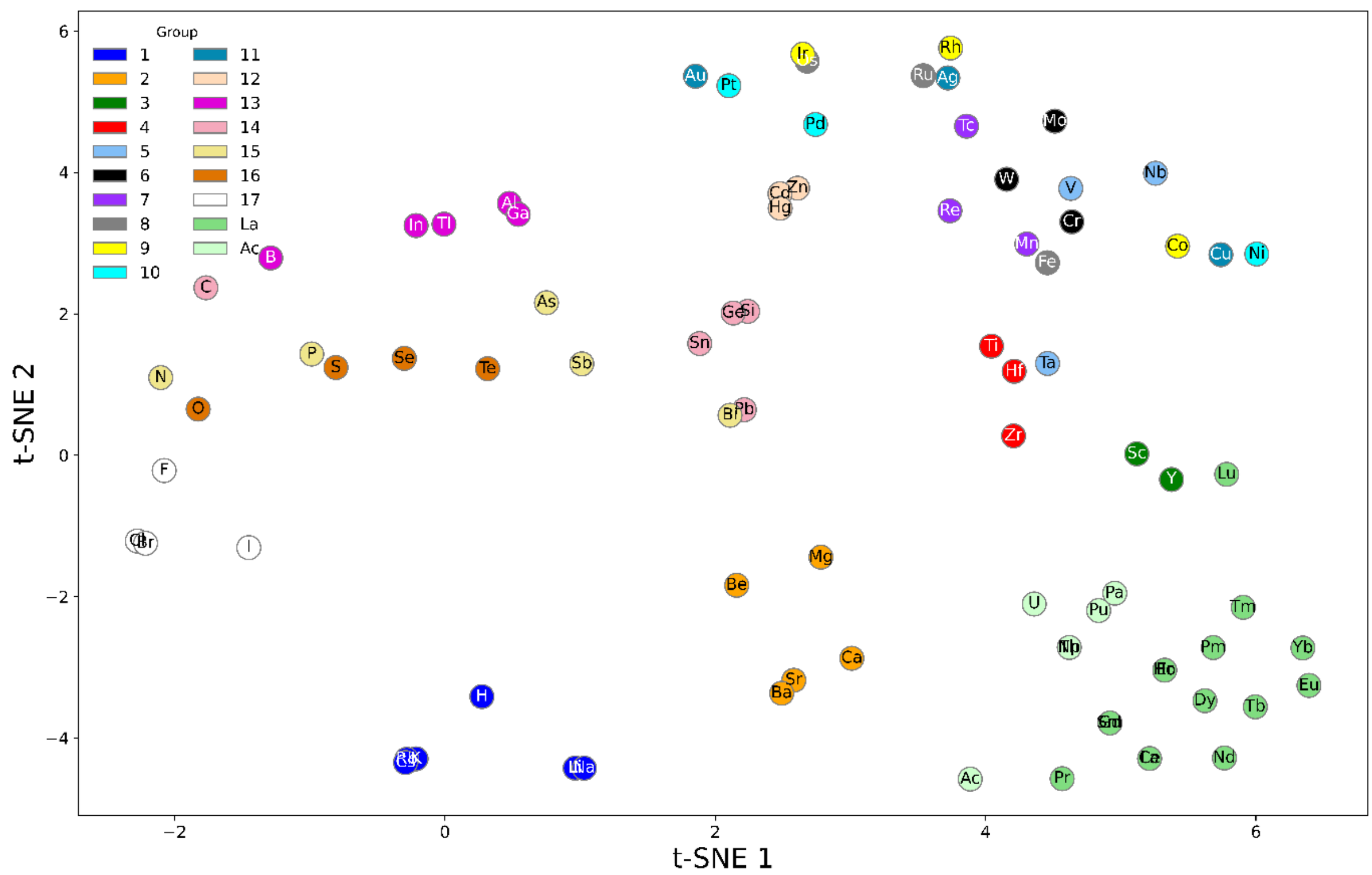

Figure S8. Two-dimensional t-SNE projection of the original CGCNN descriptor.

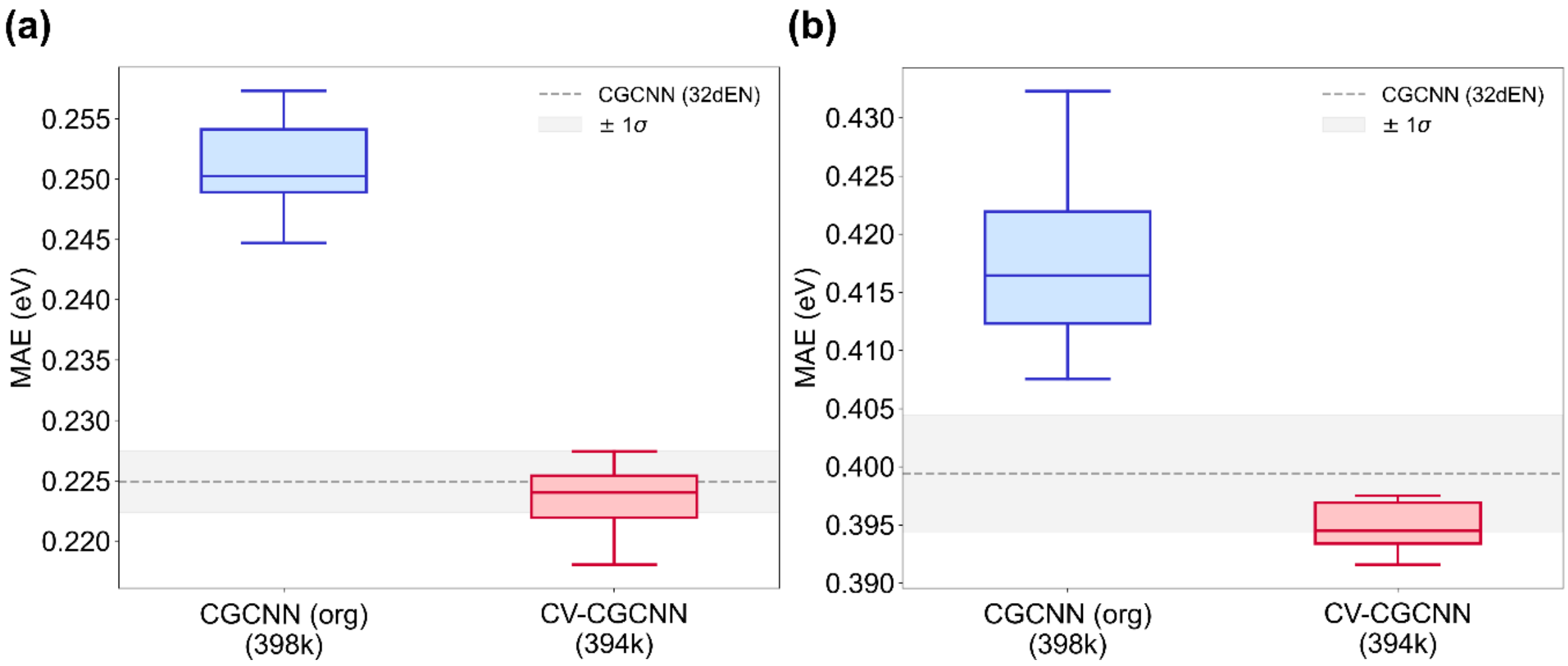

Figure S9. Prediction performance of CGCNN and CV-CGCNN for (a) band gap and (b) Fermi energy.

Note S1. Calculation of pairwise distances and angles between learned elemental descriptors

For each element i, the descriptor was represented as a d-dimensional vector $\chi_i \in \mathbb{R}^d$. For each pair (i, j), the Euclidean distance was computed as

$$D_{ij} = \left(\chi_i - \chi_j\right)^2$$

and the pairwise angle was computed from the cosine similarity as

$$\theta_{ij} = \cos^{-1} \frac{\chi_i \cdot \chi_j}{|\chi_i||\chi_j|}$$

These metrics quantify the separation and directional relationship between elemental descriptors, respectively. For the reproducibility analysis in Fig. S7, they were computed for descriptors obtained from different random seeds and compared across runs.

Note S2. Detailed description of CV-CGCNN

1) Complex functions

Various forms of nonlinear activation functions have been investigated in complex-valued neural networks. Trabelsi *et al.* reported that applying nonlinear functions by treating the real and imaginary parts as separate channels, rather than imposing strong constraints on the phase, leads to improved performance.[1] Following this approach, we applied the sigmoid linear unit (SiLU) and sigmoid functions independently to the real and imaginary components as two separate channels in this study.

$$\mathbb{C}SiLU = SiLU\big(Re(z)\big) + i\, SiLU(Im(z))$$

$$\mathbb{C}Sigmoid = Sigmoid\big(Re(z)\big) + i\, Sigmoid(Im(z))$$

In addition, complex batch normalization and complex global mean pooling were implemented by applying the corresponding operations independently to the real and imaginary components.

2) Details of feature-wise linear modulation (FiLM)

According to Mulliken theory, electronegativity is closely related to electron affinity (EA) and ionization energy (IE). Based on this relationship, we generated complex-valued embeddings by modulating each dimension of the 32-dimensional electronegativity descriptor ($\chi_{ML}^{(32)}$) using EA and IE through a FiLM scheme.[2]

For each atom i, the extracted $\chi_{ML}^{(32)}$ vector is denoted as $\boldsymbol{e}_i \in \mathbb{R}^{32}$.

First, EA and IE are independently transformed into FiLM parameters:

$$(\boldsymbol{\gamma}_i^{EA}, \boldsymbol{\beta}_i^{EA}) = f_{EA}(EA_i), \quad (\boldsymbol{\gamma}_i^{IE}, \boldsymbol{\beta}_i^{IE}) = f_{IE}(IE_i)$$

where $\boldsymbol{\gamma}$ and $\boldsymbol{\beta}$ are learnable FiLM coefficients, and f_{EA} and f_{IE} are linear transformation functions. The real and imaginary components of the embedding are then defined as

$$\boldsymbol{e}_i^{real} = \boldsymbol{\gamma}_i^{EA} \odot \boldsymbol{e}_i + \boldsymbol{\beta}_i^{EA}, \quad \boldsymbol{e}_i^{imag} = \boldsymbol{\gamma}_i^{IE} \odot \boldsymbol{e}_i + \boldsymbol{\beta}_i^{IE},$$

where $\odot$ denotes element-wise multiplication. Finally, the complex-valued embedding for atom i is constructed as

$$\boldsymbol{z}_i = \boldsymbol{e}_i^{real} + i\, \boldsymbol{e}_i^{imag}, \quad \boldsymbol{z}_i \in \mathbb{C}^{32}.$$

When complex-valued embeddings are generated using only EA or IE, the training process often becomes unstable due to ill-conditioned complex-valued operations. This observation highlights the importance of incorporating distinct information into the real and imaginary components of complex-valued neural networks. Accordingly, for the edge features, the real part is constructed using the conventional radial basis function (RBF), while the imaginary part uses its complementary form $1 - \mathrm{RBF}$ (for $x \leq$ radial distance), enabling the real and imaginary channels to encode complementary information about the central atom and its neighbors.

The use of $\chi_{ML}^{(32)}$ with FiLM-based complex embedding and the proposed custom edge features each leads to an improvement of approximately 3.5% in prediction performance. These results demonstrate that encoding complementary information in the real and imaginary components is beneficial for effective learning in complex-valued neural networks.

3) Details of Complex-Valued Operation

A complex-valued linear transformation is generally defined as

$$z' = Wz, \qquad W = W_{real} + i\, W_{imag}, \qquad z = x_{real} + i\, x_{imag}.$$

where *z* is a complex vector and *W* is the complex weight matrix, and subscripts *real* and *imag* represent the real and imaginary components of complex vector or matrix, respectively.
By rewriting this operation in the real-valued space, the transformation can be expressed as

$$\begin{pmatrix} z'_{real} \\ z'_{imag} \end{pmatrix} = \begin{pmatrix} W_{real} & -W_{imag} \\ W_{imag} & W_{real} \end{pmatrix} \begin{pmatrix} z_{real} \\ z_{imag} \end{pmatrix}.$$

Different sign conventions in the real-valued block matrix, such as

$$\begin{pmatrix} W_{real} & W_{imag} \\ -W_{imag} & W_{real} \end{pmatrix}, \quad \begin{pmatrix} W_{real} & W_{imag} \\ W_{imag} & -W_{real} \end{pmatrix}, \quad \begin{pmatrix} W_{real} & W_{imag} \\ W_{imag} & W_{real} \end{pmatrix}, \quad \ldots$$

can all be transformed into each other by a simple diagonal matrix

$$P = \begin{pmatrix} \pm I & 0 \\ 0 & \pm I \end{pmatrix}$$

applied to the input or output. Since these transformations are invertible linear operations, all such sign choices are mathematically equivalent, and the specific block sign convention does not affect the expressive power or learning capability of the complex-valued linear layer.

References

1) Trabelsi, C.; Bilaniuk, O.; Zhang, Y.; Serdyuk, D.; Subramanian, S.; Santos, J. F.; Mehri, S.; Rostamzadeh, N.; Bengio, Y.; Pal, C. J. *Deep Complex Networks.* **arXiv** *2017*, **arXiv:1705.09792.**

2) Perez, E.; Strub, F.; de Vries, H.; Dumoulin, V.; Courville, A. *FiLM: Visual Reasoning with a General Conditioning Layer.* **arXiv** *2017*, **arXiv:1709.07871.**
